# The April Number

**Published:** 1889-05

**Authors:** 


The April number having been out long enough for our readers to examine it, they will readily understand that the great care required to avoid errors caused the delay in its appearance.

